# Pembrolizumab-associated primary hypothyroidism followed by central hypothyroidism: a case report

**DOI:** 10.3389/fonc.2026.1978296

**Published:** 2026-10-15

**Authors:** Jiawei Sun, Chunnuo Wang, Zhanzhan Sun, Wei Li

**Affiliations:** 1 Department of Nephrology, Cixi People’s Hospital, Wenzhou Medical University, Ningbo, Zhejiang, China; 2 Department of Medical Imaging (Radiology), Cixi People’s Hospital, Wenzhou Medical University, Ningbo, Zhejiang, China; 3 Department of Endocrinology, Cixi People Hospital Medical Health Group (Cixi People Hospital), Ningbo, Zhejiang, China; 4 Department of Endocrinology, Ningbo Medical Center Lihuili Hospital, Zhejiang, China

**Keywords:** Hashimoto’s thyroiditis, hypothyroidism, PD-1 inhibitor, pembrolizumab, pituitary hypothyroidism

## Abstract

**Introduction:**

PD-1 inhibitors are widely used anticancer drugs and frequently trigger immune-related adverse events, with thyroid dysfunction as the most prevalent. Suspected isolated central hypothyroidism emerging during PD-1 inhibitor therapy has rarely been documented.

**Case presentation:**

A 56-year-old man received pembrolizumab for esophageal squamous cell carcinoma. Before treatment, he had pre-existing Hashimoto’s thyroiditis with low TT4 and normal TSH. He developed painless thyroiditis manifesting as transient thyrotoxicosis followed by primary hypothyroidism. Shortly after hypothyroidism was pharmacologically controlled, TSH progressively declined to the lower limit of detection, accompanied by low TT4. Pituitary MRI showed pituitary gland thinning without focal structural abnormalities. Integrating the clinical course, central hypothyroidism was considered. Given altered upper gastrointestinal anatomy after esophagectomy, split-dose levothyroxine was administered, and the patient’s FT4 levels remained stable.

**Conclusion:**

This rare sequential evolution of thyroid dysfunction may represent an uncommon endocrine immune-related adverse event of immune checkpoint inhibitors.

## Introduction

Immunotherapy represents a novel treatment paradigm for malignant tumors. In recent years, immune checkpoint inhibitors (ICIs), including programmed death-1 (PD-1) inhibitors and programmed death ligand-1 (PD-L1) inhibitors, have been increasingly used as a new class of antitumor agents. However, these agents can cause immune-related adverse events (irAEs)-autoimmune end-organ damage secondary to checkpoint inhibitor use. Common endocrine irAEs include hypophysitis, thyroid dysfunction, diabetes mellitus, and adrenal insufficiency (1, 2). Among these, PD-1 inhibitors are most frequently associated with thyroid dysfunction (1). Hypothyroidism induced by PD-1 inhibitor therapy is commonly encountered in clinical practice, with reported incidences ranging from 7.0% to 8.5% for PD-1 monotherapy and up to 13.2% for combination regimens (3, 4), and typically results from destructive thyroiditis. However, central hypothyroidism secondary to primary thyroid dysfunction has not been previously reported.

Here, we report a patient with pre-existing Hashimoto’s thyroiditis presenting with low TT4 and normal TSH prior to pembrolizumab. The changes in thyroid function occurred post exposure to pembrolizumab have been unfolded in the case report.

## Case report

A 56-year-old male was diagnosed with middle thoracic esophageal squamous cell carcinoma in April 2022. A health examination in 2019 had revealed mild thyroid dysfunction: total triiodothyronine (TT3) was measured at 1.25 nmol/L (reference, 0.92-2.79), total thyroxine (TT4) at 51 nmol/L (reference, 58.1-140.6), and thyroid-stimulating hormone (TSH) at 0.938 mIU/L (reference, 0.55-4.78). Levels of thyroid peroxidase antibody (TPOAb) and thyroglobulin antibody (TgAb) were significantly elevated at 1385 IU/mL (reference, <60) and 105 IU/mL (reference, <60), respectively. Ultrasound findings of the thyroid were suggestive of Hashimoto’s thyroiditis. No intervention was undertaken. Pre-chemotherapy thyroid function tests reaffirmed this pattern of low TT3 and TT4 alongside a normal TSH (**Table 1**). The patient was not receiving thyroid hormone replacement therapy.

**Table 1:** Serial thyroid function and autoantibody profiles during pembrolizumab treatment.

Time (week post-pembrolizumab, date)	Thyroid hormone intervention	TT3 (nmol/L)	TT4 (nmol/L)	FT3 (pmol/L)	FT4 (pmol/L)	TSH (mIU/L)	TPOAb (IU/mL)	TgAb (IU/mL)
Pre-treatment, 2022-04-11	None	0.93↓	53.87↓	–	11.47	0.82	–	–
Week 3, 2022-05-06	None	5.27↑	164.49↑	–	–	<0.004↓	–	–
Week 6, 2022-05-27	L-T4 50 μg qd	0.62↓	14.31↓	–	–	6.032↑	–	–
Week 18, 2022-08-22	L-T4 100 μg qd	<0.15↓	<3.9↓	<0.31↓	<1.29↓	119.96↑	–	–
Week 23, 2022-09-22	L-T4 100 μg qd	0.39↓	31.2↓	1.21↓	0.46↓	71.86↑	240.7↑	551.7↑
Week 28, 2022-10-27	L-T4 100 μg qd	0.67↓	68.0	2.76↓	1.29↓	2.36	213↑	347↑
Week 31, 2022-11-21	L-T4 125 μg qd	0.85↓	64.1	3.37	1.55↓	0.06↓	361↑	202↑
Week 41, 2023-01-27	L-T4 125 μg qd	0.76↓	45.8↓	2.90↓	1.09↓	0.08↓	228↑	419↑
Week 46, 2023-03-06	L-T4 150 μg qd	1.20	77.70	6.51↑	23.67↑	0.013↓	–	–

Serial thyroid function and autoantibody profiles during pembrolizumab therapy (initiated 15 April 2022). Time points show weeks post-pembrolizumab and sampling dates. Thyroid hormone intervention denotes daily levothyroxine (L-T4) dose at sampling. Reference ranges: TT3 1.01–2.48 nmol/L; TT4 55.34–160.88 nmol/L; FT3 3.28–6.47 pmol/L; FT4 7.98–16.02 pmol/L; TSH 0.35–4.94 mIU/L; TPOAb <34 IU/mL; TgAb <115 IU/mL. ↑ = above, ↓ = below reference range.

On April 15, 2022, the patient initiated preoperative neoadjuvant therapy with pembrolizumab (200 mg), nab-paclitaxel (400 mg), and carboplatin (400 mg) administered every 21 days. Three weeks after the first dose of the PD-1 inhibitor, on May 6, 2022, the patient developed thyrotoxicosis (TT3 5.27 nmol/L; TT4 164.49 nmol/L; TSH <0.004 mIU/L), consistent with pembrolizumab-induced painless thyroiditis. Three weeks thereafter (May 27, 2022), repeat thyroid function testing revealed progression to hypothyroidism (TT3 0.62 nmol/L; TT4 14.31 nmol/L; TSH 6.032 mIU/L). The patient received levothyroxine sodium tablets at an initial dose of 50 μg once daily, with good adherence; doses were taken on an empty stomach and separated from other concomitant medications. On July 7, 2022, the patient underwent robot-assisted thoracoscopic radical esophagectomy, followed by adjuvant chemotherapy with pembrolizumab, paclitaxel, and carboplatin. Serial monitoring revealed progressive hypothyroidism, with TSH rising to 119.96 mIU/L, prompting an increase in the levothyroxine dose to 100 μg daily. After completion of the final chemotherapy cycle on 23 September 2022, pembrolizumab was continued. With levothyroxine replacement, the patient’s thyroid function gradually stabilized.

At week 31 of pembrolizumab therapy (21 November 2022), follow-up tests showed incipient declines in TSH and TT4 compared with prior measurements: TT4 stayed within normal limits, while TSH dropped below the reference range. Subsequent tests showed substantial synchronous reductions in TSH and TT4 (**Table 1**). In March 2023, he was referred to endocrinology for asthenia. Physical examination showed no goiter or thyroid tenderness, although myxedema was present. During this period, assessment of the pituitary-adrenal axis showed a mildly elevated morning (8:00) ACTH level of 49.80 pg/mL (reference, 0.00-46.00), with a normal 8:00 cortisol level of 179.64 ng/mL (reference, 52.70-224.50) and preserved diurnal rhythm. Follicle-stimulating hormone (FSH) was mildly elevated at 13.02 IU/L (reference, 1.40-8.10), whereas other pituitary-gonadal hormones were within normal limits (**Table 2**). Liver and kidney function, glucose and electrolytes were unremarkable. Thyroid ultrasonography revealed bilateral coarse echogenicity, suggestive of Hashimoto’s thyroiditis. Magnetic resonance imaging (MRI) of the pituitary gland demonstrated a thinned gland with a height of 3.5 mm and central concavity. Signal intensity was homogeneous, with no deviation of the pituitary stalk or evidence of a space-occupying lesion. Contrast-enhanced imaging showed uniform enhancement without any abnormalities (**Figure 1**).

**Table 2:** Pituitary-target gland axis assessment.

Parameter	Patient value	Reference range
Cortisol (8:00)	179.64 ng/mL	52.70-224.50 ng/mL
Cortisol (16:00)	65.4 ng/mL	34.40-167.60 ng/mL
ACTH (8:00)	49.80 pg/mL	0.00-46.00 pg/mL
ACTH (16:00)	24.10 pg/mL	-
FSH	13.02 IU/L	1.40-8.10 IU/L
LH	3.06 IU/L	1.50-9.30 IU/L
Estradiol	89 pmol/L	<146 pmol/L
Prolactin	150.0 mIU/L	45.0-375.0 mIU/L
Progesterone	1.44 nmol/L	0.89-3.88 nmol/L
Testosterone	9.33 nmol/L	6.51-23.74 nmol/L

**Figure 1:**
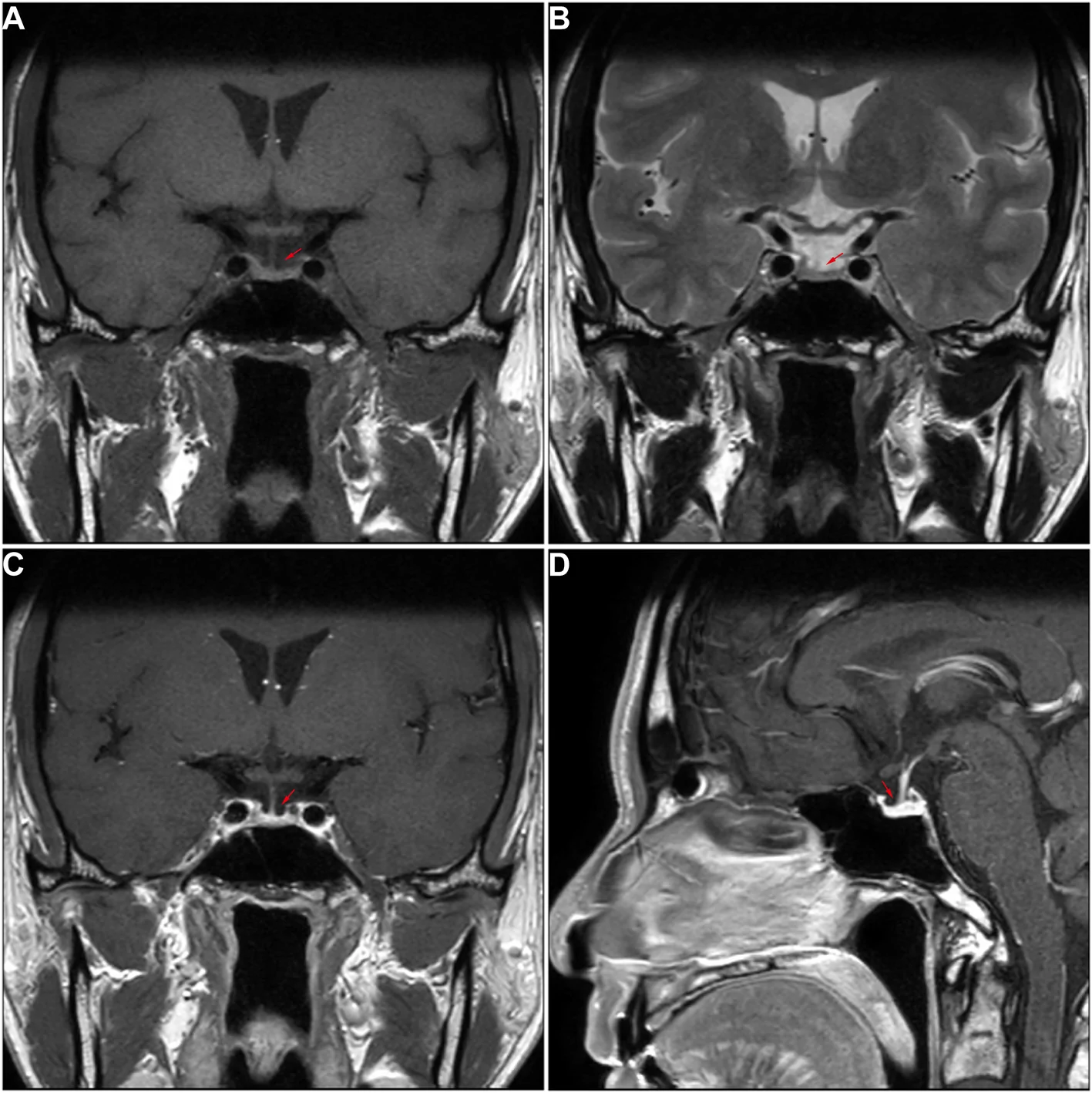
MRI findings of the pituitary gland. **(A, B)** Coronal unenhanced T1WI and T2WI through the midline show a thinned gland with central concavity (height approximately 3.5 mm), homogeneous signal intensity, and no pituitary stalk deviation, sellar floor depression, or optic chiasm compression. **(C, D)** Coronal and sagittal contrast-enhanced T1WI demonstrate marked homogeneous enhancement of the pituitary gland without any abnormal enhancing lesion. Red arrows in **(A–D)** denote the thinned, centrally concave pituitary gland.

In summary, the patient was diagnosed with pembrolizumab-induced painless thyroiditis, primary hypothyroidism, and isolated central hypothyroidism (ICH). Levothyroxine was gradually titrated to 175 μg daily. In August 2023, follow-up testing demonstrated low TT4 (**Supplementary Table S1**). The patient was 1.55 m tall and weighed 50 kg, and these 175 μg daily doses exceeded the conventional replacement dose for men after total thyroidectomy in our region. Given the patient’s history of esophagectomy, levothyroxine bioavailability may have been altered; thus, the regimen was adjusted to 50 μg three times daily. On subsequent follow-up, TSH remained persistently low, with only mild elevations in FT4. Urinary cortisol, serum sodium, and sexual function assessments were unremarkable. Thyroid ultrasonography demonstrated bilateral thyroid atrophy with coarse, heterogeneous parenchymal echotexture.

The patient completed planned neoadjuvant and postoperative antitumor therapy with acceptable tolerability and manageable treatment-related adverse events. He showed good follow-up adherence and provided written informed consent for publication of these data.

## Discussion

Our case describes a rare irAE phenotype in a patient with pre-existing Hashimoto’s thyroiditis receiving neoadjuvant pembrolizumab for esophageal squamous cell carcinoma. The patient developed painless thyroiditis complicated by primary hypothyroidism requiring levothyroxine replacement; central hypothyroidism emerged after biochemical euthyroidism was stabilized. Thyroid irAEs are among the most common endocrine toxicities of PD-1 inhibitors, with reported incidences ranging from 3.2% to 10.1% in patients treated with pembrolizumab (5, 6). However, reports of central hypothyroidism arising after painless thyroiditis and primary hypothyroidism in the same PD-1 inhibitor-treated patient remain scarce.

Thyroid dysfunction is the most common immune checkpoint inhibitor (ICI)-related endocrine adverse event and is often permanent (7). ICI-induced thyroid dysfunction occurs more frequently with anti-PD-1 monoclonal antibodies, with a mean onset time of six weeks after the first treatment (8). A meta-analysis reported the incidences of hypothyroidism as 3.8% for anti-CTLA-4, 7.0% for anti-PD-1, and 3.9% for anti-PD-L1 therapy, with the highest rate (13.2%) observed in patients receiving nivolumab plus ipilimumab (4). Thyrotoxicosis typically precedes hypothyroidism. The underlying pathophysiology is thought to be immune-mediated acute inflammation followed by thyroid destruction, classically presenting with hypovascularity on ultrasound, low radioiodine uptake, and absence of TSH receptor antibodies. The abundant expression of PD-L1 and PD-L2 on thyroid follicular cells may explain the gland’s particular susceptibility.

Our patient had markedly elevated baseline TPOAb and TgAb, indicating pre-existing autoimmune thyroiditis. Patients with positive baseline TPOAb or TgAb have a significantly higher risk of developing thyroid irAEs (9–12). PD-1 inhibitors may disrupt thyroid immune homeostasis by activating systemic T-cell responses (11). However, the relationship is incompletely understood, as some patients with thyroid irAEs are TPOAb-negative, implying antibody-independent pathways (5). Nevertheless, elevated baseline TPOAb or a history of hypothyroidism predicts worse outcomes, warranting close follow-up.

The clinical course of the patient can be divided broadly into three phases. In phase 1, prior to pembrolizumab initiation, the patient had Hashimoto’s thyroiditis accompanied by reduced TT4 with TSH within the normal reference range, suggesting possible coexistence of mild central hypothyroidism. In phase 2, after pembrolizumab-related painless thyroiditis, severe hypothyroidism developed and responded favorably to levothyroxine. While TSH increased, the magnitude of this compensatory TSH rise was less than expected relative to the accompanying reduction in FT4, suggestive of a suboptimal pituitary TSH secretory response. In the late phase, TT4 and TSH normalized whereas T3 and FT3 were low. In view of the patient’s profound debilitation, non-thyroidal illness syndrome was suspected. Phase 3 represents the most unusual phase of this case. Shortly after hypothyroidism was well controlled, the patient developed a progressive TSH decline to the lower limit of detection, alongside reduced TT3 and TT4 levels. Based on the chronological sequence of disease progression and treatment, we speculate that on the background of pre-existing Hashimoto’s thyroiditis and mild central hypothyroidism, the patient developed delayed PD-1 inhibitor-related pituitary injury, leading to impaired TSH axis function.

The interval from the onset of progressive TSH decline to the completion of systematic pituitary function evaluation spanned nearly 15 weeks. Accordingly, we were unable to capture early transient radiological alterations such as pituitary enlargement that may have occurred at the acute phase of pituitary injury; only late-stage pituitary atrophy was identified. Transient pituitary enlargement can emerge rapidly and resolve within weeks in the setting of ICI-mediated pituitary injury, meaning such acute-phase imaging features are easily missed with delayed radiological assessment (13).

PD-1 inhibitor-related immune hypophysitis represents a rare immune-related adverse event, with a reported incidence of 0.5%–1% (14). Existing studies have shown that PD-1-associated hypophysitis typically develops in the late phase of immunotherapy, with a median onset of 26 weeks. It predominantly affects elderly male individuals; pituitary imaging may be unremarkable, and affected patients may be asymptomatic of headache (15). These characteristics are consistent with the present case. However, almost all previously documented cases are complicated by adrenocortical insufficiency, and hypophysitis presenting exclusively with isolated TSH deficiency has not been described (16, 17). Over the 4-year follow-up period, pituitary function evaluation confirmed intact adrenocortical, gonadal, and neurohypophyseal function, with no occurrence of hyponatremia or diabetes insipidus. In the later disease course, the patient’s TSH remained persistently suppressed, indicating sustained impairment of pituitary TSH secretory capacity. Prior studies demonstrated that central hypothyroidism from ICI-related hypophysitis may show spontaneous recovery of TSH secretion in some patients and is usually well-controlled with levothyroxine replacement (18, 19). However, this feature differs from this typical pattern.

In terms of pathogenesis, PD-1 inhibitors may not represent the primary trigger of this condition but could exacerbate pre-existing lesions; mild central hypothyroidism at baseline may serve as the underlying etiology. Although complete pre-treatment pituitary function data were unavailable, the patient exhibited isolated TSH axis impairment without additional anterior pituitary dysfunction after progression to severe central hypothyroidism. This finding suggests that subclinical ICH might have existed prior to immunotherapy. Existing literature indicates that most cases of ICH are caused by genetic mutations. ICH can occasionally arise secondary to autoimmune hypophysitis, yet such patients typically present with multiple pituitary axis impairments, headache, and characteristic abnormalities on pituitary magnetic resonance imaging, which were absent in our patient.

Furthermore, the patient’s post-esophagectomy status posed a unique management challenge. For the patient’s body size, levothyroxine was titrated to a relatively high daily dose of 175 μg. This exceeded the usual local replacement range for post-thyroidectomy patients, yet total T4 remained mildly low. After switching to 50 μg three times daily, serial thyroid tests revealed recurrent mild FT4 elevations. Post-esophagectomy gastrointestinal remodeling may reduce levothyroxine absorption and produce variable bioavailability. Levothyroxine is absorbed mainly in the jejunum; upper gastrointestinal anatomical changes modify intestinal drug exposure and absorption efficiency (20, 21). This practical management issue is rarely addressed in literature, underscoring the critical need for personalized dosing in patients with gastrointestinal malabsorption.

This study is limited to a single retrospective case report. The TRH stimulation test was not performed to evaluate pituitary reserve, no biopsy was obtained, and baseline pre-treatment pituitary MRI was lacking. Nonetheless, the observed biochemical trajectory, imaging findings, and management difficulties provide valuable clinical insights despite the absence of confirmatory tests.

## Conclusion

Thyroiditis is a well-recognized immune-related adverse event associated with PD-1 inhibitors. This case reports a pembrolizumab-treated patient with esophageal squamous cell carcinoma and underlying Hashimoto’s thyroiditis, demonstrating a rare sequential evolution from primary hypothyroidism due to painless thyroiditis to central hypothyroidism. Pituitary function also warrants monitoring during PD 1 inhibitor treatment. Furthermore, this case underscores the need for individualized levothyroxine dosing in patients with altered upper gastrointestinal anatomy after esophagectomy.

## Data Availability

The original contributions presented in the study are included in the article/**Supplementary Material**. Further inquiries can be directed to the corresponding authors.

## References

[1] Chang L-S Barroso-Sousa R Tolaney SM Hodi FS Kaiser UB Min L . Endocrine toxicity of cancer immunotherapy targeting immune checkpoints. Endocr Rev. (2018) 40:17–65. doi: 10.1210/er.2018-00006 30184160 PMC6270990

[2] Byun DJ Wolchok JD Rosenberg LM Girotra M . Cancer immunotherapy—immune checkpoint blockade and associated endocrinopathies. Nat Rev Endocrinol. (2017) 13:195–207. doi: 10.1038/nrendo.2016.205 28106152 PMC5629093

[3] de Filette J Andreescu CE Cools F Bravenboer B Velkeniers B . A systematic review and meta-analysis of endocrine-related adverse events associated with immune checkpoint inhibitors. Horm Metab Res Horm Stoffwechselforschung Horm Metab. (2019) 51:145–56. doi: 10.1055/a-0843-3366 30861560

[4] Barroso-Sousa R Barry WT Garrido-Castro AC Hodi FS Min L Krop IE . Incidence of endocrine dysfunction following the use of different immune checkpoint inhibitor regimens: A systematic review and meta-analysis. JAMA Oncol. (2018) 4:173–82. doi: 10.1001/jamaoncol.2017.3064 28973656 PMC5838579

[5] Delivanis DA Gustafson MP Bornschlegl S Merten MM Kottschade L Withers S . Pembrolizumab-induced thyroiditis: Comprehensive clinical review and insights into underlying involved mechanisms. J Clin Endocrinol Metab. (2017) 102:2770–80. doi: 10.1210/jc.2017-00448 28609832 PMC5546861

[6] Wang F Shi X Yu X Yang Y . Immune checkpoint inhibitor-induced isolated adrenocorticotropic hormone deficiency: A systematic review. Front Endocrinol. (2024) 15. doi: 10.3389/fendo.2024.1326684 38318292 PMC10838970

[7] Husebye ES Castinetti F Criseno S Curigliano G Decallonne B Fleseriu M . Endocrine-related adverse conditions in patients receiving immune checkpoint inhibition: An ESE clinical practice guideline. Eur J Endocrinol. (2022) 187:G1–G21. doi: 10.1530/EJE-22-0689 36149449 PMC9641795

[8] Duan L Wang L Wang H Si X Zhang L Liu X . Clinical diagnosis and treatment of immune checkpoint inhibitors‐related endocrine dysfunction. Thorac Cancer. (2020) 11:1099–104. doi: 10.1111/1759-7714.13347 32043816 PMC7113039

[9] Okada N Iwama S Okuji T Kobayashi T Yasuda Y Wada E . Anti-thyroid antibodies and thyroid echo pattern at baseline as risk factors for thyroid dysfunction induced by anti-programmed cell death-1 antibodies: A prospective study. Br J Cancer. (2020) 122:771–7. doi: 10.1038/s41416-020-0736-7 32009131 PMC7078193

[10] Osorio JC Ni A Chaft JE Pollina R Kasler MK Stephens D . Antibody-mediated thyroid dysfunction during T-cell checkpoint blockade in patients with non-small-cell lung cancer. Ann Oncol Off J Eur Soc Med Oncol. (2017) 28:583–9. doi: 10.1093/annonc/mdw640 27998967 PMC5834017

[11] Zhao P Li J Yu L Ma W Zhao T . Clinical manifestations and risk factors of immune-related thyroid adverse events in patients treated with PD-1 inhibitors: A case-control study. Front Immunol. (2025) 16:1581057. doi: 10.3389/fimmu.2025.1581057 40356893 PMC12066586

[12] Brilli L Danielli R Campanile M Secchi C Ciuoli C Calabrò L . Baseline serum TSH levels predict the absence of thyroid dysfunction in cancer patients treated with immunotherapy. J Endocrinol Invest. (2021) 44:1719–26. doi: 10.1007/s40618-020-01480-6 33367977 PMC8285335

[13] Chamorro-Pareja N Faje AT Miller KK . Pituitary complications of checkpoint inhibitor use. Endocrinology. (2024) 165:bqae084. doi: 10.1210/endocr/bqae084 39001874

[14] Wright JJ Powers AC Johnson DB . Endocrine toxicities of immune checkpoint inhibitors. Nat Rev Endocrinol. (2021) 17:389–99. doi: 10.1038/s41574-021-00484-3 33875857 PMC8769055

[15] Di Stasi V La Sala D Cozzi R Scavuzzo F De Geronimo V Poggi M . Immunotherapy-related hypophysitis: A narrative review. Cancers. (2025) 17:436. doi: 10.3390/cancers17030436 39941803 PMC11815778

[16] Xiao Y Jiang D Wang S Fu L Ye L Zheng E . Clinical characteristics and comparative analysis of PD-1/PD-L1 inhibitor-induced pituitary dysfunction versus other pituitary disorders. Sci Rep. (2025) 15:21353. doi: 10.1038/s41598-025-05563-3 40594483 PMC12214842

[17] Kobayashi T Iwama S Yasuda Y Okada N Okuji T Ito M . Pituitary dysfunction induced by immune checkpoint inhibitors is associated with better overall survival in both malignant melanoma and non-small cell lung carcinoma: A prospective study. J Immunother Cancer. (2020) 8:e000779. doi: 10.1136/jitc-2020-000779 32606047 PMC7328763

[18] van der Leij S Suijkerbuijk KPM van den Broek MFM Valk GD Dankbaar JW van Santen HM . Differences in checkpoint-inhibitor-induced hypophysitis: Mono- versus combination therapy induced hypophysitis. Front Endocrinol. (2024) 15. doi: 10.3389/fendo.2024.1400841 39135626 PMC11317883

[19] Min L Hodi FS Giobbie-Hurder A Ott PA Luke JJ Donahue H . Systemic high-dose corticosteroid treatment does not improve the outcome of ipilimumab-related hypophysitis: A retrospective cohort study. Clin Cancer Res. (2015) 21:749–55. doi: 10.1158/1078-0432.CCR-14-2353 25538262 PMC4334697

[20] Incidence, diagnosis and management of malabsorption following oesophagectomy: a systematic review, in: Journal of gastrointestinal surgery. Springer Nature Link. Available online at: https://link.springer.com/article/10.1007/s11605-022-05323-y (Accessed August 2, 2026). 10.1007/s11605-022-05323-y 35484473

[21] Liu H Li W Zhang W Sun S Chen C . Levothyroxine: Conventional and novel drug delivery formulations. Endocr Rev. (2023) 44:393–416. doi: 10.1210/endrev/bnac030 36412275 PMC10166268

