# Supplementary material for: Pembrolizumab-associated primary hypothyroidism followed by central hypothyroidism: a case report
Source: Front Oncol. 2026 Oct 15;16:1978296. doi: 10.3389/fonc.2026.1978296 (PMC13716603; doi:10.3389/fonc.2026.1978296)
Supplement: Supplementary file 1 [file Supplementaryfile1.docx]

**Supplemental Figure Legends**

**Figure S1.** Serial thyroid function tests and levothyroxine dose adjustments after pembrolizumab exposure. Week 0 corresponds to the time before the first pembrolizumab infusion. RAMIE: robot-assisted thoracoscopic radical esophagectomy. Grey shading denotes daily levothyroxine dose.

**Figure S2.** During more than two years of follow-up, contrast-enhanced brain MRI findings. (A) No obvious mass lesion. (B) Cerebrospinal fluid signal in the pituitary fossa.

**Supplementary Table S1.** Early follow-up thyroid function tests (local hospital)

| **Date** | **Thyroid hormone intervention** | **TT3 (ng/mL)** | **TT4 (nmol/L)** | **FT3 (pg/mL)** | **FT4 (ng/dL)** | **TSH (mIU/L)** |
| --- | --- | --- | --- | --- | --- | --- |
| 2023-4 | L-T4 175 μg qd | 0.81 | 62.1↓ | 3.39 | 1.61 | 0.01↓ |
| 2023-6 | L-T4 175 μg qd | 0.82 | 74.6 | 3.26 | 1.74↑ | <0.01↓ |
| 2023-8 | L-T4 50 μg tid | 3.68 | 65.7↓ | 3.68 | 1.84↑ | <0.01↓ |
| 2023-9 | L-T4 50 μg tid | 0.82 | 75.5 | 3.43 | 1.97↑ | <0.01↓ |
| 2023-11 | L-T4 50 μg tid | 0.80 | 75 | 3.57 | 1.99↑ | <0.01↓ |
| 2024-1 | L-T4 50 μg tid | 0.93 | 87.7 | 3.65 | 2.11↑ | 0.02 |
| 2024-4 | L-T4 50 μg tid | 0.65↓ | 85.5 | 2.9 | 2.08↑ | 0.01↓ |
| 2024-7 | L-T4 50 μg tid | 0.82 | 86.3 | 3.17 | 1.91↑ | <0.01↓ |
| 2024-9 | L-T4 50 μg tid | 0.89 | 81 | 2.58 | 2.17↑ |  |
| 2024-12 | L-T4 50 μg tid | 1.01 | 71.8 | 3.38 | 1.57 | <0.01↓ |
| 2025-2 | L-T4 50 μg tid | 0.84 | 82.2 | 3.2 | 1.76↑ | <0.01↓ |

Reference ranges were updated during follow-up. Reference intervals: TT3, 0.80–2.00 ng/mL; TT4, 66–181 nmol/L; FT3, 2–4.4 pg/mL; FT4, 0.93–1.7 ng/dL; TSH, 0.27–4.2 mIU/L. ↑ = above reference interval; ↓ = below reference interval.

**Supplementary Table S2**. Late follow-up thyroid function tests (local hospital)

| **Date** | **Thyroid hormone intervention** | **TT3 (ng/mL)** | **TT4 (ug/dL)** | **FT3 (pmol/L)** | **FT4 (pmol/L)** | **TSH (mIU/L)** |
| --- | --- | --- | --- | --- | --- | --- |
| 2025-05 | L-T4 50 μg tid | 0.83↓ | 89.7 | 5.33 | 20.31 | <0.01↓ |
| 2025-8 | L-T4 50 μg tid | 0.84 | 7.22 | 6.04 | 22.20↑ | <0.01↓ |
| 2025-10 | L-T4 50 μg tid | 0.85 | 8.64 | 5.33 | 20.31 | <0.01↓ |
| 2025-11 | L-T4 50 μg tid | 0.89 | 9.11 | 7.07 | 21.04 |  |
| 2026-1 | L-T4 50 μg tid | 0.93 | 9.76 | 5.58 | 22.09↑ | <0.01↓ |
| 2026-4 | L-T4 50 μg tid | 0.80 | 5.99 | 3.9 | 22.03↑ | <0.01↓ |
| 2026-7 | L-T4 50 μg tid | 0.92 | 8.29 | 3.88 | 20.47 | <0.01↓ |

Reference ranges were updated during follow-up. Reference intervals: TT3, 0.8–1.9 nmol/L; TT4, 5–13 nmol/L; FT3, 3.5–7.0 pmol/L; FT4, 10–22 pmol/L; TSH, 0.35–4.75 mIU/L. ↑ = above reference interval; ↓ = below reference interval.
